# Oblique lumbar interbody fusion versus minimally invasive transforaminal lumbar interbody fusion for the treatment of degenerative disease of the lumbar spine: a systematic review and meta-analysis

**DOI:** 10.1007/s10143-023-02009-0

**Published:** 2023-04-29

**Authors:** Yun-lu Wang, Xi-yong Li, Lun Liu, Song-feng Li, Peng-fei Han, Xiao-dong Li

**Affiliations:** 1 Department of Orthopaedics, The Second People’s Hospital of Changzhi City, Changzhi, People’s Republic of China; 2 Graduate School, Changzhi Medical College, Changzhi, People’s Republic of China; 3 Department of Orthopaedics, Heping Hospital Affiliated to Changzhi Medical College, Changzhi, People’s Republic of China

**Keywords:** Oblique lumbar interbody fusion, Minimally invasive transforaminal lumbar interbody fusion, Degenerative disease of the lumbar spine, Treatment, Meta-analysis

## Abstract

This meta-analysis compared the efficacy of oblique lumbar interbody fusion (OLIF) and minimally invasive transforaminal lumbar interbody fusion (MIS-TLIF) in the treatment of lumbar degenerative diseases. A computer search for the published literature on OLIF and MIS-TLIF for the treatment of lumbar degenerative diseases in the PubMed, Web of Science, Embase, CINAHL, MEDLINE, Cochrane Library, and other databases was performed, from which 522 related articles were retrieved and 13 were finally included. Two reviewers independently extracted data from the included studies and analyzed them using RevMan 5.4. The quality of the studies was assessed using the Cochrane systematic analysis and the Newcastle–Ottawa scale. Meta-analysis showed that the blood loss [95% confidence intervals (CI) (− 121.01, − 54.56), $P<0.001$], hospital stay [95% CI (− 1.98, − 0.85), $P<0.001$], postoperative fusion rate [95%CI (1.04, 3.60), $P=0.04$], postoperative disc height [95% CI (0.50, 3.63), $P=0.01$], and postoperative foraminal height [95% CI (0.96, 4.13), $P=0.002$] were all better in the OLIF group; however, the complication rates were significantly lower in the MIS-TLIF group [95% CI (1.01, 2.06), $P=0.04$]. However, there were no significant differences between the two in terms of surgery time, patient satisfaction, or postoperative functional scores. The OLIF group had the advantages of lower blood loss, a shorter hospital stay, a higher postoperative fusion rate, and better recovery of the disc and foraminal heights, whereas MIS-TLIF had a relatively lower complication rate.

## Introduction

Lumbar degenerative diseases are a series of diseases mainly caused by the gradual degeneration of the intervertebral disc with age, including lumbar disc herniation with or without spondylolisthesis, lumbar spinal stenosis, lumbar degenerative spondylolisthesis, scoliosis associated with lumbar degenerative spondylolisthesis, and discogenic low back pain [1]. Conservative treatment is the first choice of treatment for most patients, and surgery is recommended when conservative treatment fails. Lumbar fusion surgery is a widely used procedure to treat spinal disorders and has traditionally been performed using the posterior lumbar interbody fusion (PLIF) or transforaminal lumbar interbody fusion (TLIF) methods [2]. Studies have shown that damage from paraspinal dissection and sustained stretching can lead to ischemia, denervation, and lumbar muscle dysfunction, resulting in chronic pain and poor postoperative clinical outcomes [3]. Consequently, new surgical modalities are being developed. Foley et al. [4] introduced MIS-TLIF in 2003, which can directly decompress neural structures without extensive dissection of the paraspinal muscles and ligaments, thereby avoiding delayed spinal instability due to excessive muscle and soft tissue dissection. Studies have shown that MIS-TLIF minimizes soft tissue destruction and spinal segment instability compared to TLIF surgical modalities, resulting in less paraspinal muscle damage, less perioperative blood loss and pain, a shorter hospital stay, and a faster postoperative recovery [5]. But MIS-TLIF is performed through a smaller surgical access, and the intraoperative field of view is limited, which inevitably increases the surgery time [6]. Oblique lumbar interbody fusion (OLIF), proposed by Silvestre et al. [7], is a retroperitoneal approach between the psoas muscle and the great abdominal vessels. Access to the surgical segment or intervertebral space through the natural anatomical space allows direct access to the intervertebral disc, complete debridement of the intervertebral disc, and the placement of a larger cage. The advantages of OLIF are that it achieves indirect decompression, corrects coronal and sagittal imbalances, reduces paraspinal muscle trauma, and minimizes blood loss. It is worth mentioning that vascular injury is a potential intraoperative risk factor for OLIF because it is usually performed in areas adjacent to segmental vessels and major abdominal vessels [8]. However, determining which of the two is better remains the focus of attention in the treatment of lumbar degenerative diseases. Therefore, this meta-analysis aimed to compare and evaluate the postoperative efficacy of OLIF and MIS-TLIF in the treatment of lumbar degenerative disease and the differences between the two surgical modalities based on currently published literature.

## Materials and methods

### Surgical techniques

#### OLIF

The patient was placed in the right lateral decubitus position, and a 4 cm left-to-right incision was made over a two-finger width anterior to the anterior superior iliac crest. A blunt dissection of the external oblique abdominals, internal oblique and transversus abdominis muscles, and the peritoneum and fascia of the transversus abdominis muscle was performed. After reaching between the left iliac artery and the psoas major muscle, the instrument was used to pull the psoas major muscle to the dorsal side and the iliac artery to the ventral side. After adequate exposure of the target disc, discectomy, debridement of the cartilaginous endplates, and implantation of the fusion device were performed.

#### MIS-TLIF

The patient was placed in the prone position, and the surgical plane was determined preoperatively using portable radiography. First, further TLIF was performed using a unilateral Wiltse’s paraspinal approach. The skin, soft tissue, and back muscles were pulled using a tubular retractor to expose the facet joints. Hemilaminectomy and facet joint resection were performed to decompress the nerve roots. Cages were placed in the intervertebral space after the completion of nerve decompression and endplate preparation. The surgical procedure was performed under a surgical microscope with variable magnification and focus.

### Inclusion and exclusion criteria

The inclusion criteria were as follows: (1) controlled clinical studies; (2) studies that included patients diagnosed with lumbar degenerative diseases, such as lumbar disc herniation, lumbar spinal stenosis, and lumbar spondylolisthesis, and required surgery after failure of conservative treatment; and (3) studies that used OLIF and MIS-TLIF as interventions.

The exclusion criteria were as follows: (1) nonclinical controlled studies, case reports, reviews, letters, and duplicate reports; (2) studies that included patients with deformities, spinal infections, spinal fractures, benign or malignant tumors of the spine, revision surgery of the same grade, or neck or chest lesions.

The outcome indicators were surgery time, blood loss, hospital stay, patient satisfaction, postoperative fusion rate, postoperative DH and FH, postoperative Japanese Orthopedic Association (JOA) score, postoperative Oswestry Disability Index (ODI) function score, postoperative visual analog scale (VAS) score, and complications, a total of 11 items.

### Search strategy

We searched PubMed, Web of Science, Embase, CINAHL, MEDLINE, the Cochrane Library, and other databases. Additionally, relevant studies were reviewed to expand the search. There were no restrictions on sample size, age of participants, or the language of the article. The search keywords used were OLIF, MIS-TLIF, and degenerative disease of the lumbar spine. The search strategy was ([“OLIF”] OR [“oblique lumbar interbody fusion”] OR [“MIS-TLIF”] OR [“minimally invasive transforaminal lumbar interbody fusion”] OR [“pre-psoas lateral interbody fusion”] OR [“antepsoas lateral interbody fusion”]) AND “degenerative disease of the lumbar spine”.

### Quality assessment of the included literature

Two researchers independently extracted data using a predesigned standard protocol, and disagreements were resolved by discussion until a consensus was reached or the quality of the literature was jointly assessed with a third researcher. This was strictly assessed according to the Cochrane risk-of-bias assessment criteria. At the same time, the quality of the literature was evaluated according to the Newcastle–Ottawa scale (NOS) [9], which includes three dimensions and eight items in total: four items for research object selection, one item for intergroup comparability, and results that measure three items. Except for the comparability item, which could get a maximum of two points, the other items could get a maximum of one point, and the score range was 0–9 points. The higher the overall score, the higher the quality of the study. Studies containing multiple cohorts were scored separately. Among the outcome measures, scores were specified when the follow-up time was > 1 year and the loss to follow-up rate was ≤ 5%. The NOS score was divided into three grades: low, medium, and high quality, with < 5, 5–7, and ≥ 8 points, respectively.

### Statistical analysis

This study was performed according to the Preferred Reporting Items for Systematic reviews and Meta-Analyses (PRISMA) guidelines. Meta-analysis of the extracted data was performed using RevMan 5.4 software. Continuous variables were expressed as mean differences (MD) and 95% confidence intervals (CI), and dichotomous variables were expressed as odds ratios (OR) and 95% CI. Heterogeneity determined using the *I*^2^ statistic was defined as follows: when *I*^2^ was < 50%, indicating that the heterogeneity between studies was small, a fixed-effects model was used. When *I*^2^ ≥ 50%, it indicated that the heterogeneity between studies was large, and a random-effects model was used. Currently, it is necessary to assess publication bias and conduct a sensitivity analysis to identify evidence of heterogeneity. The level of statistical significance was set at a *P* value < 0.05.

## Results

### Essential features of the included literature

**Fig. 1:**
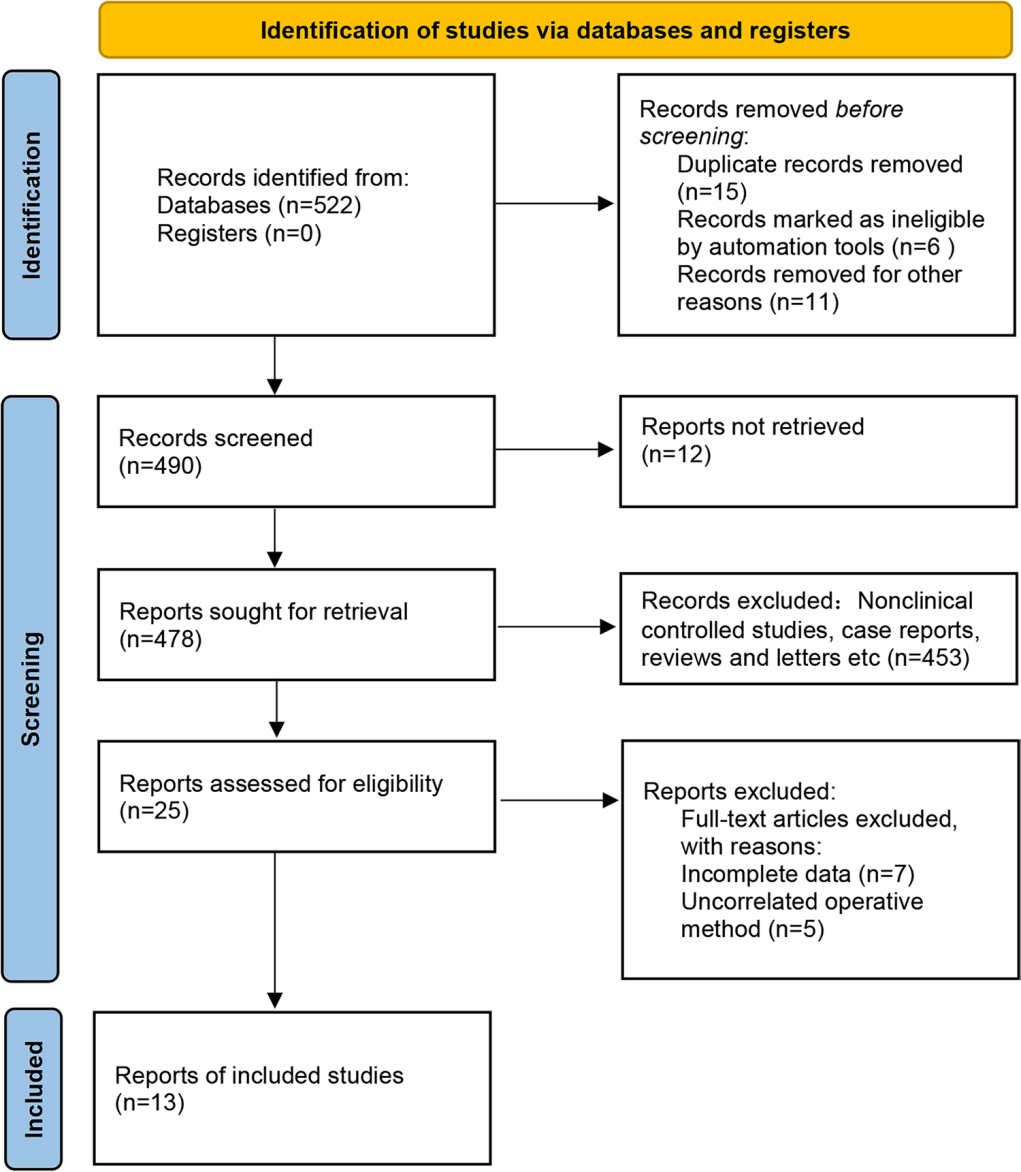
Flow diagram of study identification and selection

Based on this search strategy, 522 relevant articles were retrieved. Duplicate published studies were deleted, and by reading the titles and abstracts, nonclinical controlled studies, case reports, reviews, and letters were excluded, and 25 relevant studies were initially screened. The screening was performed after reading the full texts according to the inclusion and exclusion criteria, and a total of 13 articles were included in the final analysis. All included studies compared the baseline conditions of patients, such as age and sex, and were comparable ($P>0.05$). The literature screening process and results are shown in Fig. 1, and the basic characteristics of the included studies are listed in Table 1 [10–22].

**Table 1:** Characteristics of included literature studies

Author	Study design	Country	Year	Group	Patients	Age (years)	Gender (M/F)	Outcomes	NOS scale
Abbasi [10]	Retrospective	USA	2018	OLIF	68	54.66 ± 16.34	35/33	(1) (2) (3)	7
				MISTLIF	28	58.21 ± 8.99	10/18		
Champagne [11]	Retrospective	Canada	2019	OLIF	38	62	15/23	(11)	7
				MISTLIF	65	62	28/37		
Chandra [12]	Retrospective	India	2022	OLIF	28	52.50	9/19	(1) (2) (5) (9)	7
				MISTLIF	28	49.96	9/19		
Gao [13]	Retrospective	China	2022	OLIF	53	58.42 ± 9.98	23/30	(1) (2) (3) (9) (10) (11)	8
				MISTLIF	60	59.23 ± 11.66	28/32		
Han [14]	Retrospective	China	2021	OLIF	28	50.4 ± 16.0	12/16	(1) (2) (3) (4) (5) (6) (9) (11)	7
				MISTLIF	33	53.6 ± 13.5	15/18		
Hung [15]	Retrospective	China	2021	OLIF	21	62.33 ± 12.08	10/11	(1) (2) (3) (5) (6) (7) (9) (10) (11)	8
				MISTLIF	41	60.32 ± 13.34	28/13		
Koike [16]	Retrospective	Japan	2020	OLIF	38	72.1 ± 11.4	20/18	(1) (2) (6) (8) (10) (11)	6
				MISTLIF	48	70.1 ± 11.5	18/30		
Kotani A [17]	Retrospective	Japan	2020	OLIF	33	63.1 ± 35.45	15/18	(1) (2) (5) (8) (11)	8
				MISTLIF	38	64.7 ± 52.89	25/13		
Kotani B [18]	Retrospective	Japan	2020	OLIF	92	72.0 ± 9.9	46/46	(1) (2) (6) (8) (10) (11)	7
				MISTLIF	50	70.0 ± 11.2	17/33		
Lin [19]	Retrospective	Korea	2018	OLIF	25	64 ± 7.44	8/17	(4) (5) (11)	9
				MISTLIF	25	64 ± 10.46	8/17		
Sheng [20]	Retrospective	China	2019	OLIF	38	65.29 ± 8.88	8/30	(1) (2) (3) (4) (11)	6
				MISTLIF	55	60.62 ± 12.37	25/30		
Yingsakmongkol [21]	Retrospective	Thailand	2021	OLIF	30	63 ± 9.7	8/22	(1) (2) (3) (5) (6) (7) (9) (10) (11)	7
				MISTLIF	30	67.1 ± 5.29	6/24		
Zhu [22]	Prospective	China	2021	OLIF	68	60.2 ± 6.2	36/32	(1) (2) (3) (5) (9) (10) (11)	7
				MISTLIF	62	61.1 ± 5.3	33/29		

Outcomes: (1) surgery time, (2) blood loss, (3) hospital stay, (4) patient satisfaction, (5) postoperative fusion rate, (6) postoperative DH, (7) postoperative FH, (8) postoperative JOA score, (9) postoperative ODI function score, (10) postoperative VAS score, (11) complications

### Quality assessment of the included literature

This review included one prospective study and 12 retrospective studies. The quality of the literature was evaluated using the NOS. Among them, one study scored nine points, and three studies scored eight points, for a total of four high-quality studies. Seven studies scored seven points, two studies scored six points, nine studies were of medium quality, and no studies were of low quality.

### Outcomes

#### Comparison of perioperative indicators

**Fig. 2:**
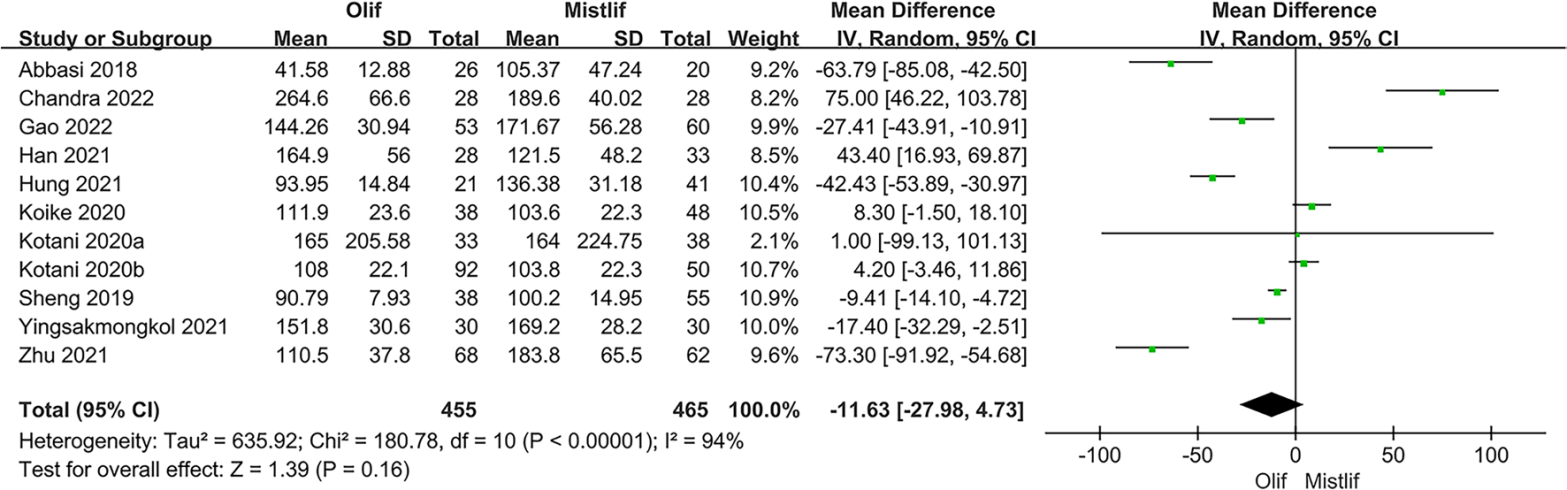
Forest plot surgery time

**Fig. 3:**
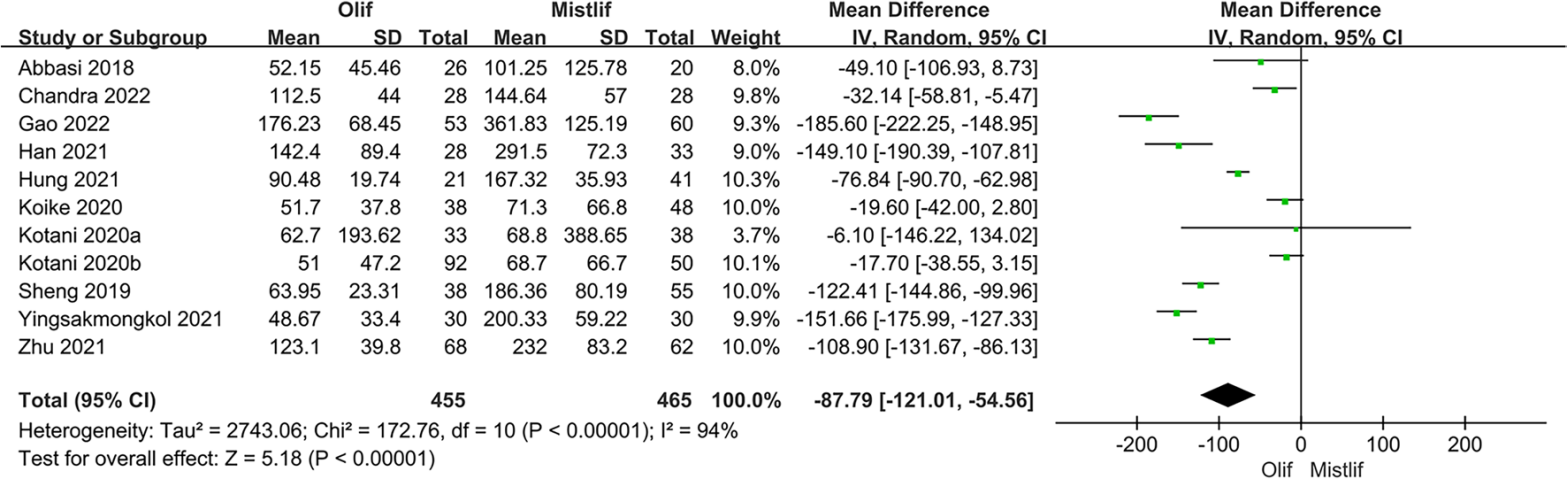
Forest plot blood loss

**Fig. 4:**
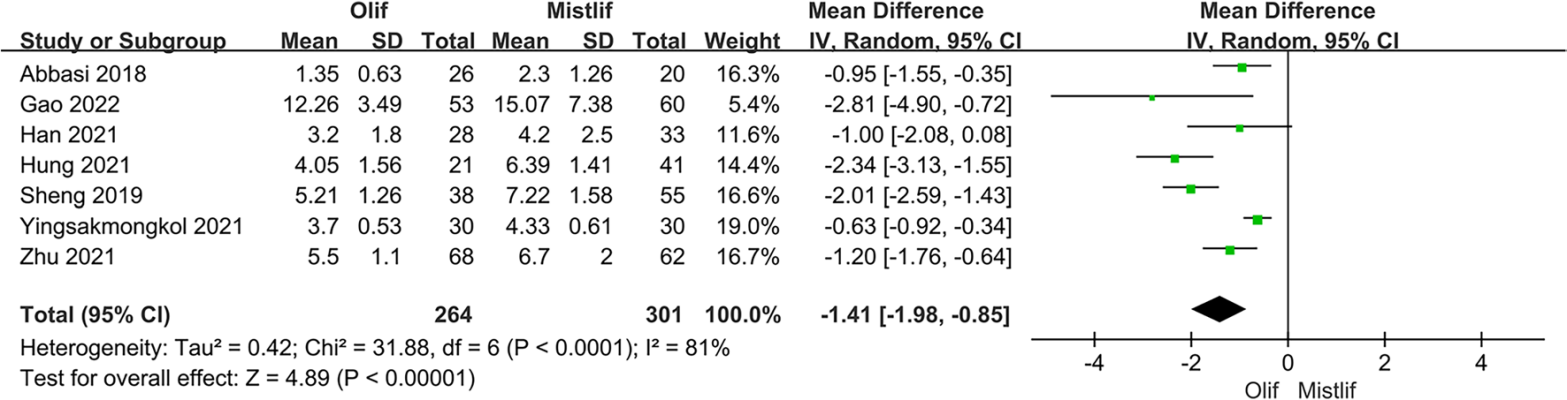
Forest plot hospital stay

Perioperative indicators included surgical time, blood loss, and length of hospital stay. Eleven studies including 920 patients compared the operative time and intraoperative blood loss between OLIF and MIS-TLIF. The results of the heterogeneity test showed that there was significant heterogeneity among the studies comparing surgery time and intraoperative blood loss (*I*^2^ = 94.0%; $P<0.001$); therefore, a random-effects model was used for the meta-analysis. The results showed that the surgery times in the two groups were similar without a statistically significant difference [95%CI (− 27.98, 4.73), $P=0.16$] (Fig. 2). In the treatment of lumbar degenerative disease, the blood loss in the OLIF group was significantly lower than that in the MIS-TLIF group [95% CI (− 121.01, − 54.56), $P<0.001$] (Fig. 3). Seven studies compared the lengths of hospital stays between patients who underwent OLIF and MIS-TLIF. The heterogeneity test showed significant heterogeneity among the studies (*I*^2^ = 81%; $P < 0.0001$), and a random-effects model was used. The overall effect results showed that the hospital stay in the OLIF group was significantly shorter than that in the MIS-TLIF group [95% CI (− 1.98, − 0.85), $P < 0.001$] (Fig. 4).

### Comparison of patient satisfaction

**Fig. 5:**
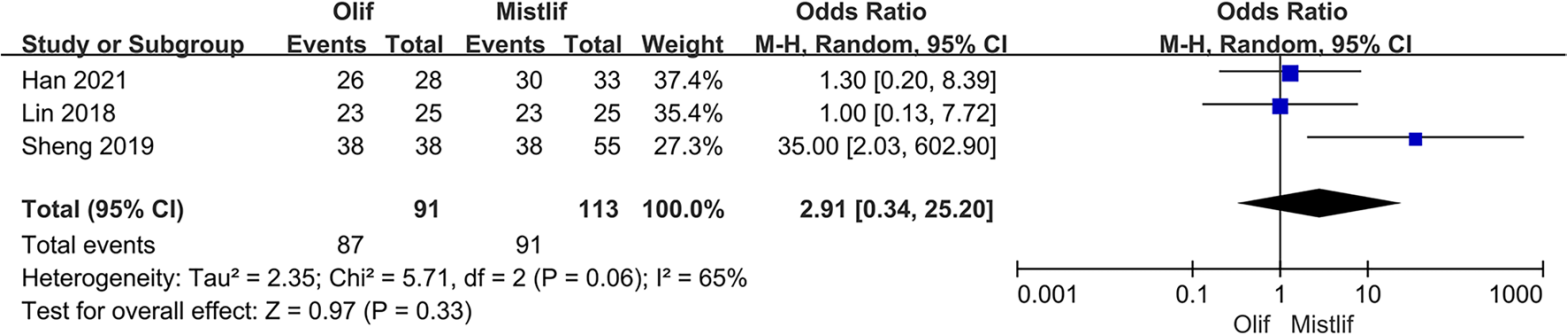
Forest plot patient satisfaction

Patient satisfaction included four levels: very satisfied, partially satisfied, partially dissatisfied, and dissatisfied. Very satisfied and partially satisfied patients were considered satisfied, whereas very dissatisfied and partially dissatisfied patients were considered dissatisfied. Three studies including 204 patients compared patient satisfaction between OLIF and MIS-TLIF. The results of the heterogeneity test showed no significant heterogeneity among the studies (*I*^2^ = 65%; $P = 0.06$). Therefore, a random-effects model was used for the meta-analysis, which showed no significant difference in patient satisfaction between the two groups [95% CI (0.34, 25.20), $P=0.33$] (Fig. 5).

### Comparison of postoperative fusion rates

**Fig. 6:**
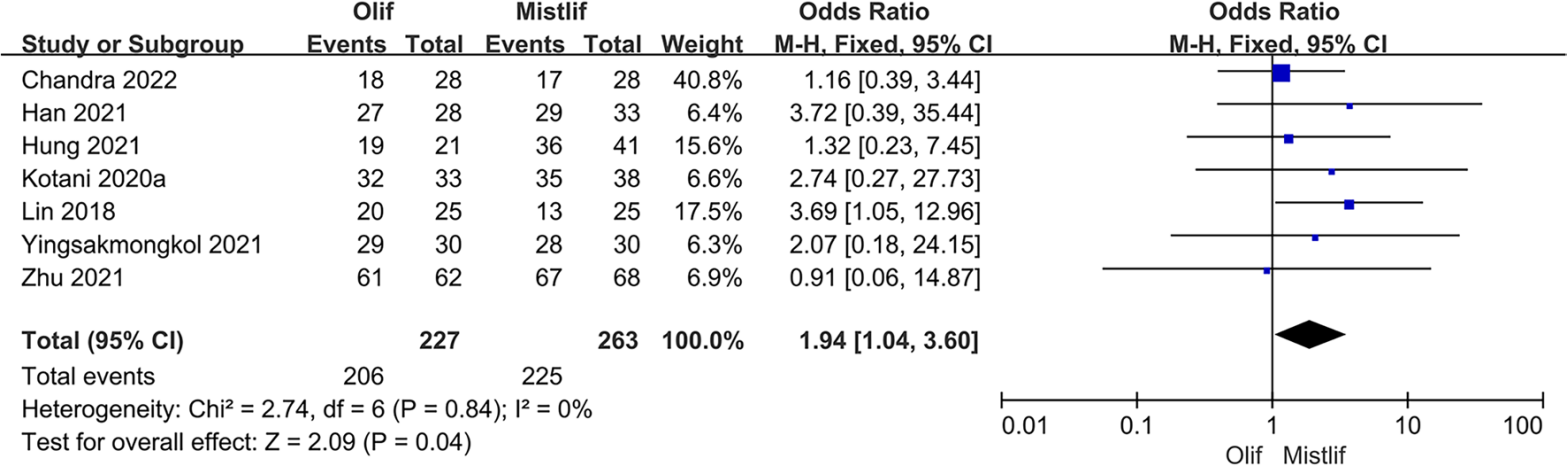
Forest plot postoperative fusion rate

The fusion rates were assessed using computed tomography and the Bridwell fusion grading system. Fusion was defined as the formation of continuous trabecular bridging bone and the lack of a gap between the vertebral end plates and the fusion apparatus in the coronal or sagittal plane. Seven studies, including 490 patients, compared the postoperative fusion rates between OLIF and MIS-TLIF. The heterogeneity test showed no significant heterogeneity among the studies (*I*^2^ = 0%; $P = 0.84$). Therefore, a fixed-effects model was used for the meta-analysis, which showed that the postoperative fusion rate in the OLIF group was 90.75% (206/227) and that in the MIS-TLIF group was 85.55% (225/263). The OLIF group had a significantly higher postoperative fusion rate than the MIS-TLIF group [95%CI (1.04, 3.60), $P=0.04$] (Fig. 6).

### Comparison of postoperative DH and FH

**Fig. 7:**
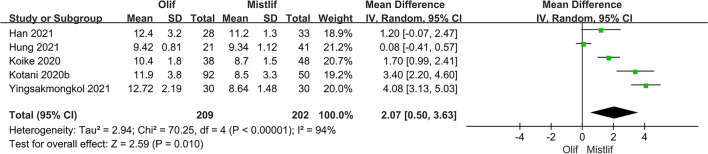
Forest plot postoperative DH

**Fig. 8:**
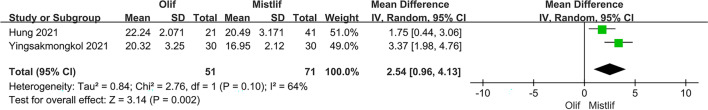
Forest plot postoperative FH

Five studies, including 411 patients, compared the postoperative DH between OLIF and MIS-TLIF. The results of the heterogeneity test showed significant heterogeneity among the studies (*I*^2^ = 94.0%, $P <0.001$). Therefore, a random-effects model was used for the meta-analysis, which showed that the DH in the OLIF group was significantly higher than that in the MIS-TLIF group [95% CI (0.50, 3.63), $P=0.01$] (Fig. 7). In terms of FH, the heterogeneity test showed significant heterogeneity among the studies (*I*^2^ = 64%; $P = 0.10$). A random-effects model showed that the FH in the OLIF group was significantly greater than that in the MIS-TLIF group [95% CI (0.96, 4.13), $P=0.002$] (Fig. 8).

### Comparison of postoperative functional scores

**Fig. 9:**
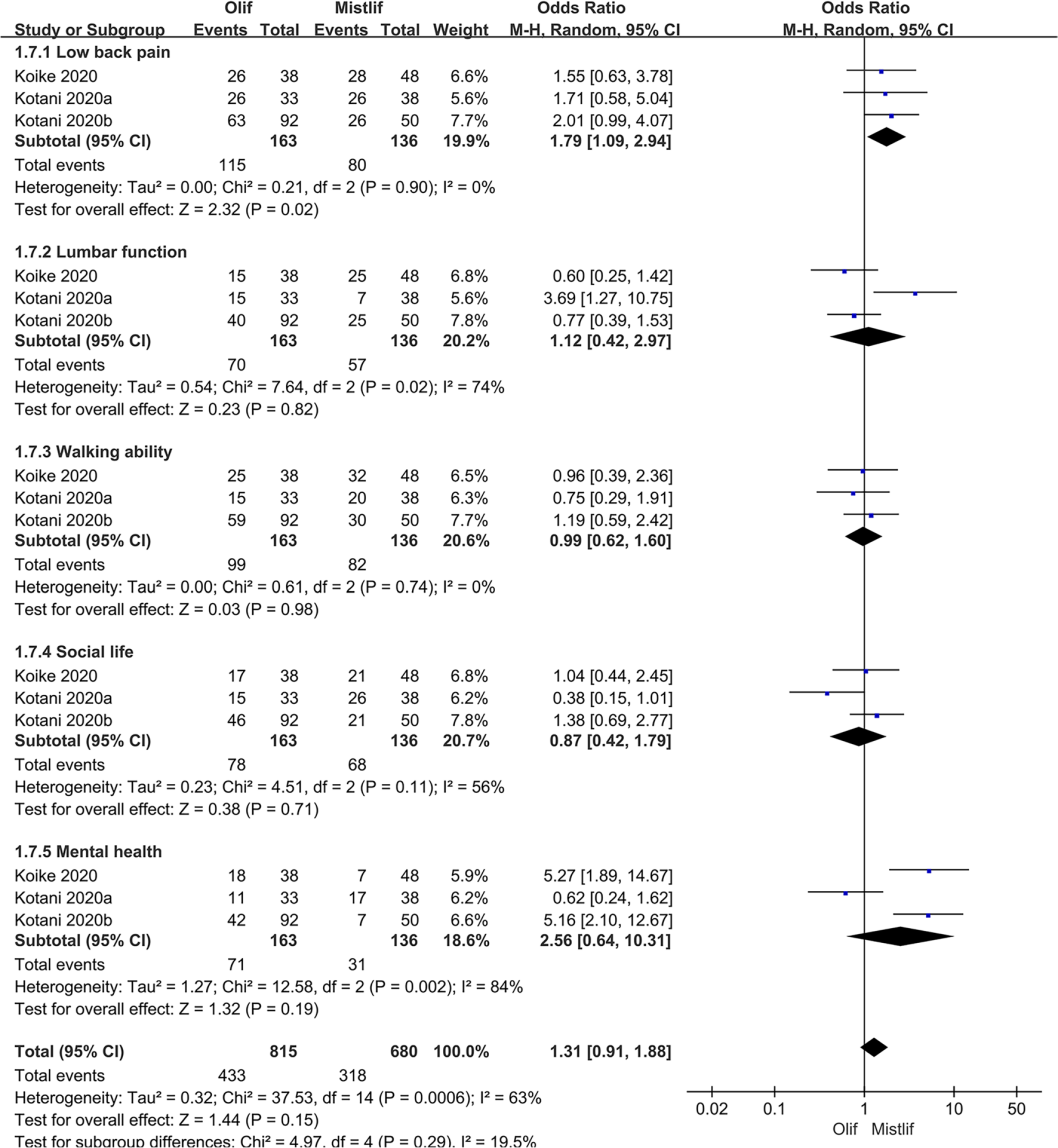
Forest plot postoperative JOA score

**Fig. 10:**
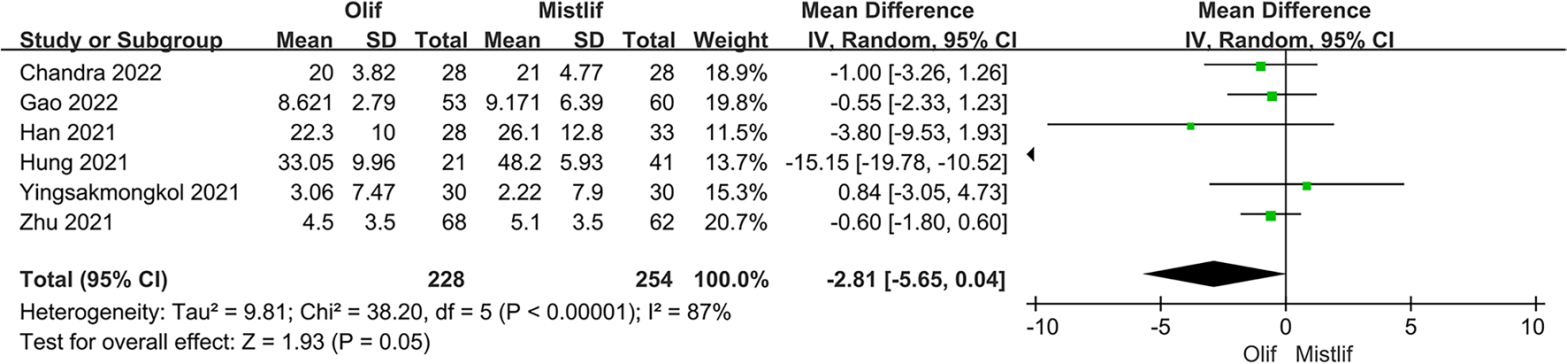
Forest plot postoperative ODI function score

**Fig. 11:**
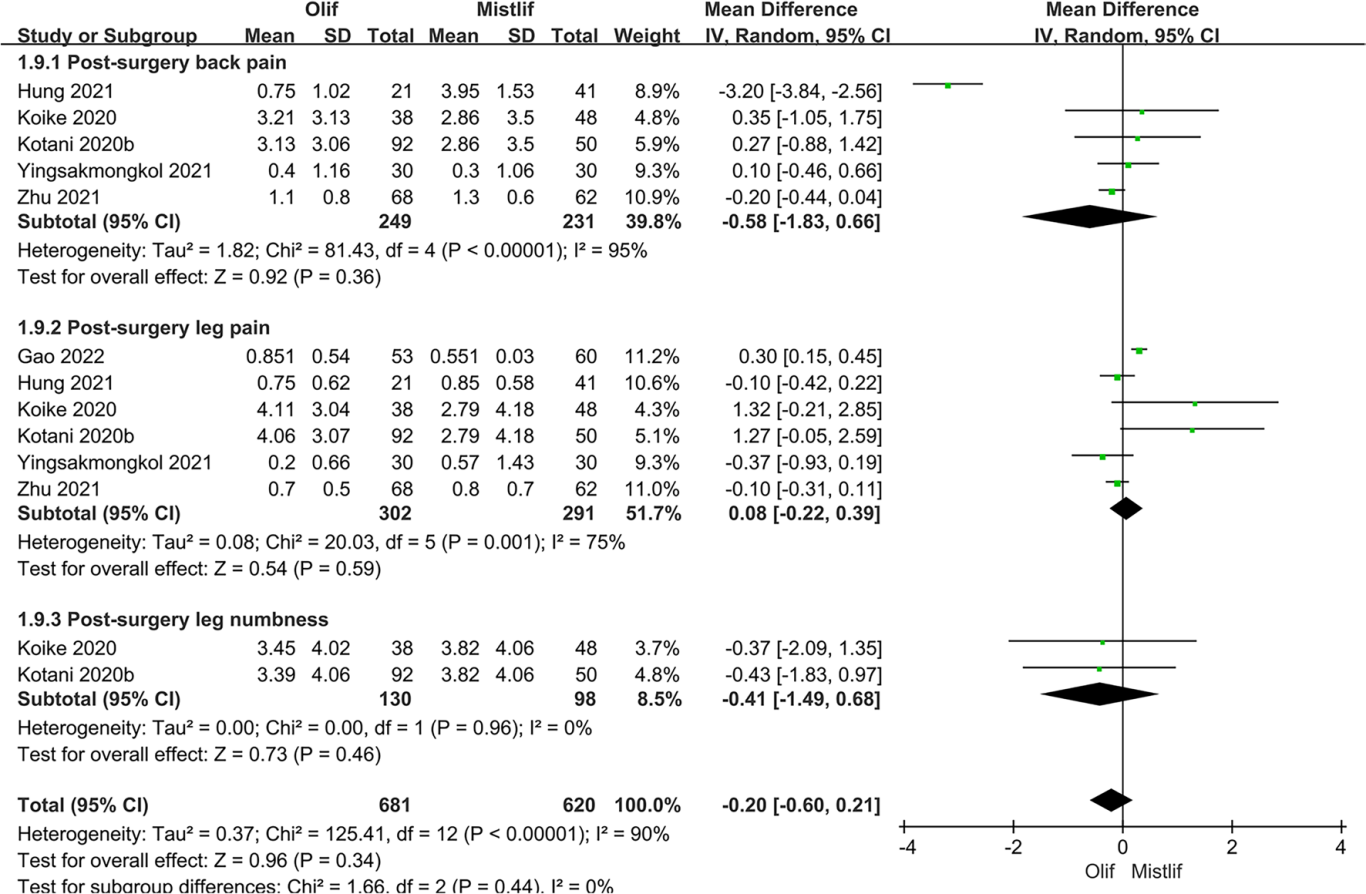
Forest plot postoperative VAS score

The postoperative functional scores included the postoperative JOA, ODI, and VAS scores, and a subgroup analysis was performed on the JOA and VAS scores. Heterogeneity analysis revealed the following results: postoperative JOA score (*I*^2^ = 63.0%; $P = 0.0006$), postoperative ODI score (*I*^2^ = 87.0%; $P <0.0001$), and postoperative VAS score (*I*^2^ = 90.0%; $P <0.0001$). There was significant heterogeneity among the studies; therefore, the random-effects model was used for classification. The results showed that the OLIF group was superior to the MIS-TLIF group in some functions, with a statistically significant difference in back pain [95%CI (1.09, 2.94), $P=0.02$] based on the JOA score (Fig. 9). The postoperative ODI [95%CI (− 5.65, 0.04), $P=0.05$] (Fig. 10) and postoperative VAS scores [95% CI (− 0.60, 0.21), $P=0.34$] (Fig. 11) were not statistically significant.

### Comparison of postoperative complications

**Fig. 12:**
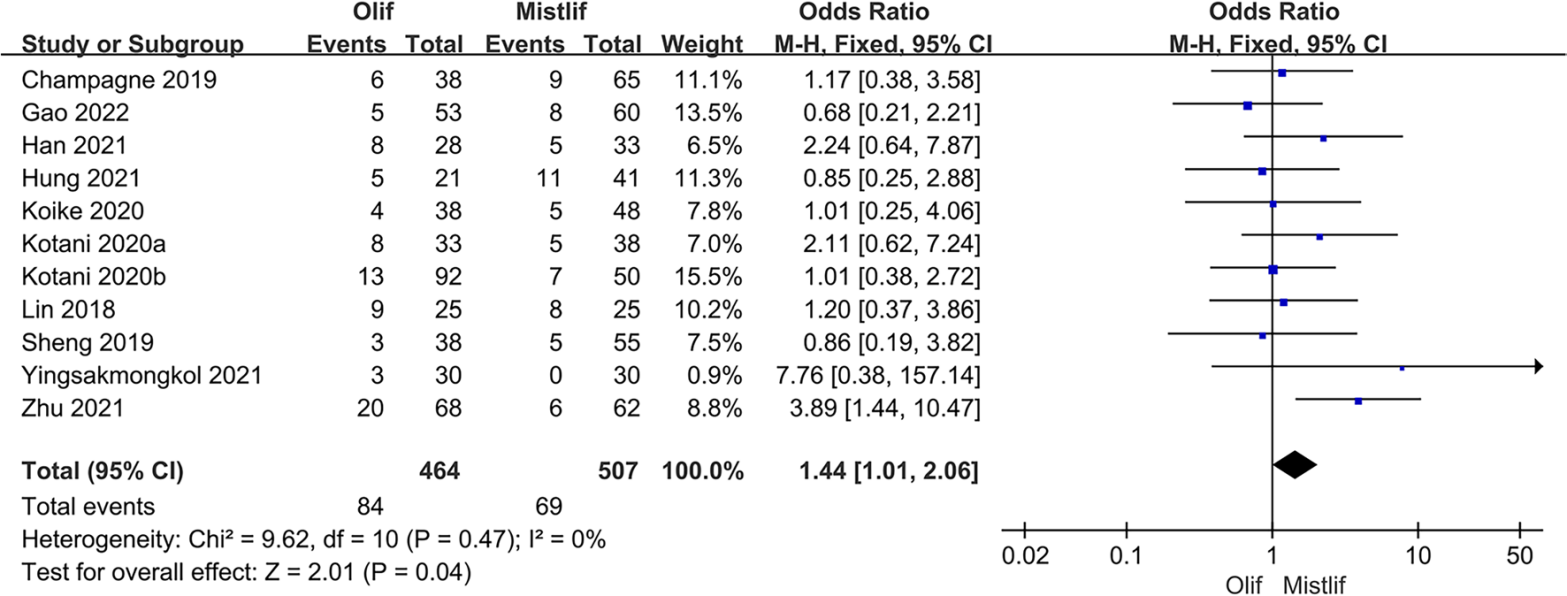
Forest plot postoperative complications

Eleven studies compared the postoperative complications of OLIF and MIS-TLIF, with an incidence of 18.10% (84/464) in the OLIF group and 13.61% (69/507) in the MIS-TLIF group. The heterogeneity test *I*^2^ = 0% showed that there was no significant heterogeneity among the studies. So, a fixed-effects model was used for the meta-analysis, which showed that the incidence of postoperative complications in the MIS-TLIF group was significantly lower than that in the OLIF group for the treatment of lumbar degenerative disease [95% CI (1.01, 2.06), $P=0.04$] (Fig. 12).

### Publication bias and sensitivity analysis

**Fig. 13:**
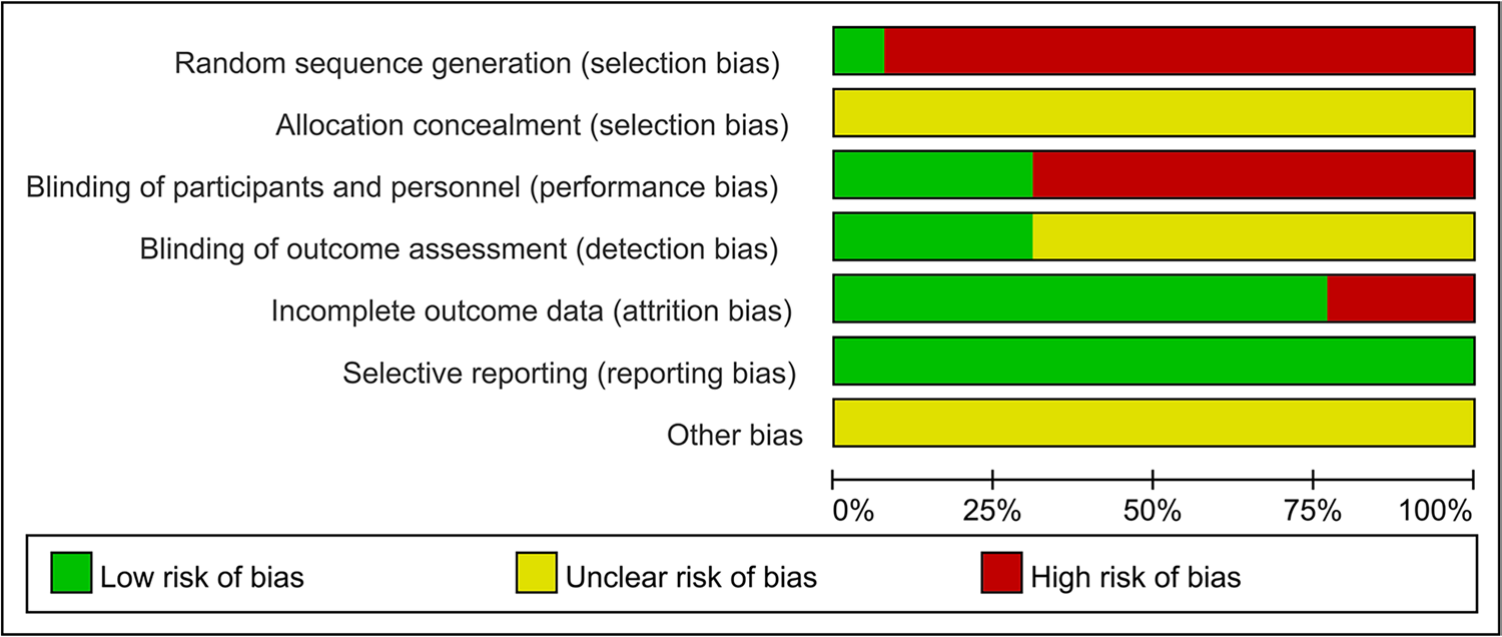
Risk of bias assessment of included studies

**Fig. 14:**
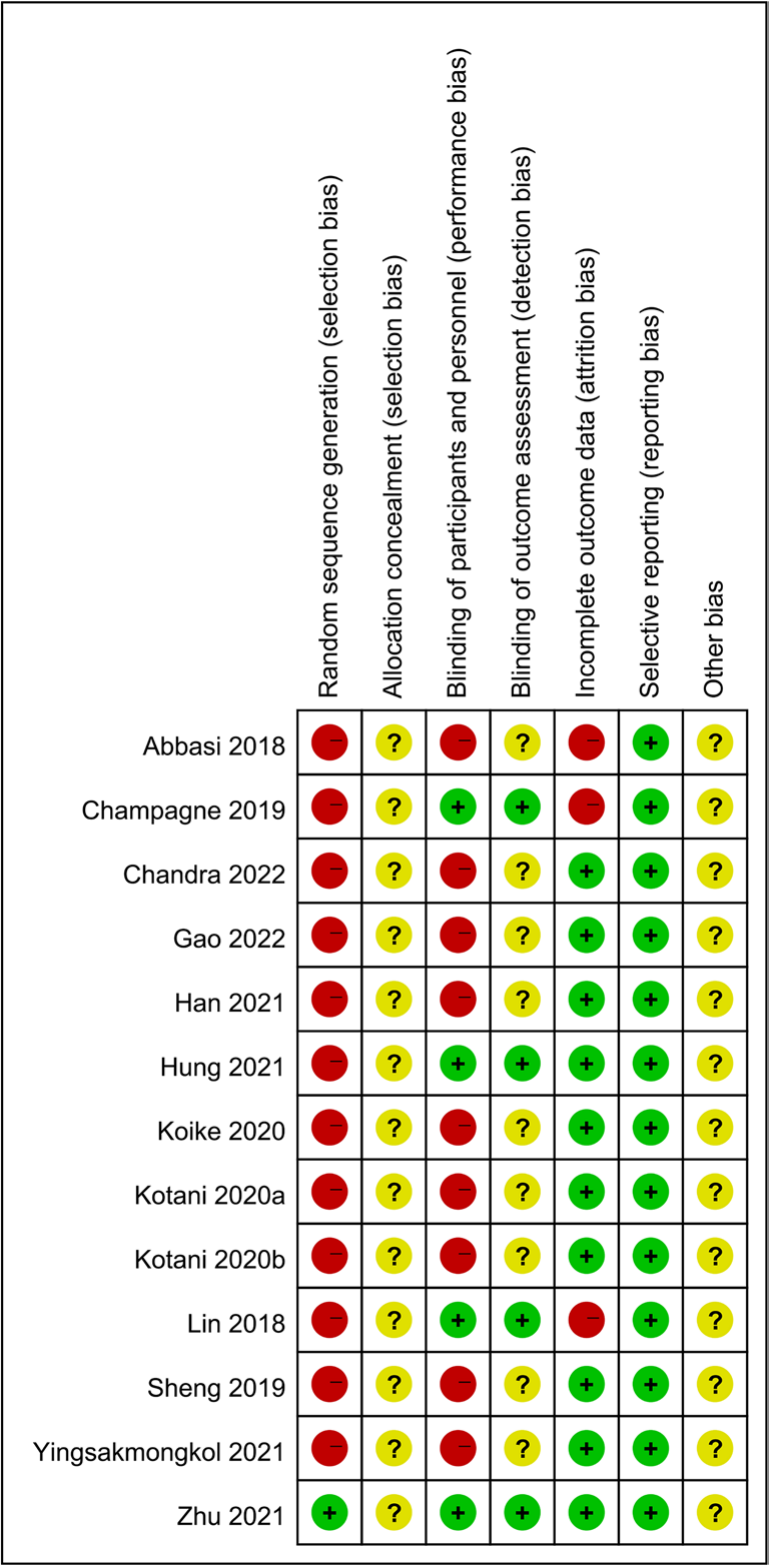
Publication bias funnel plot

**Fig. 15:**
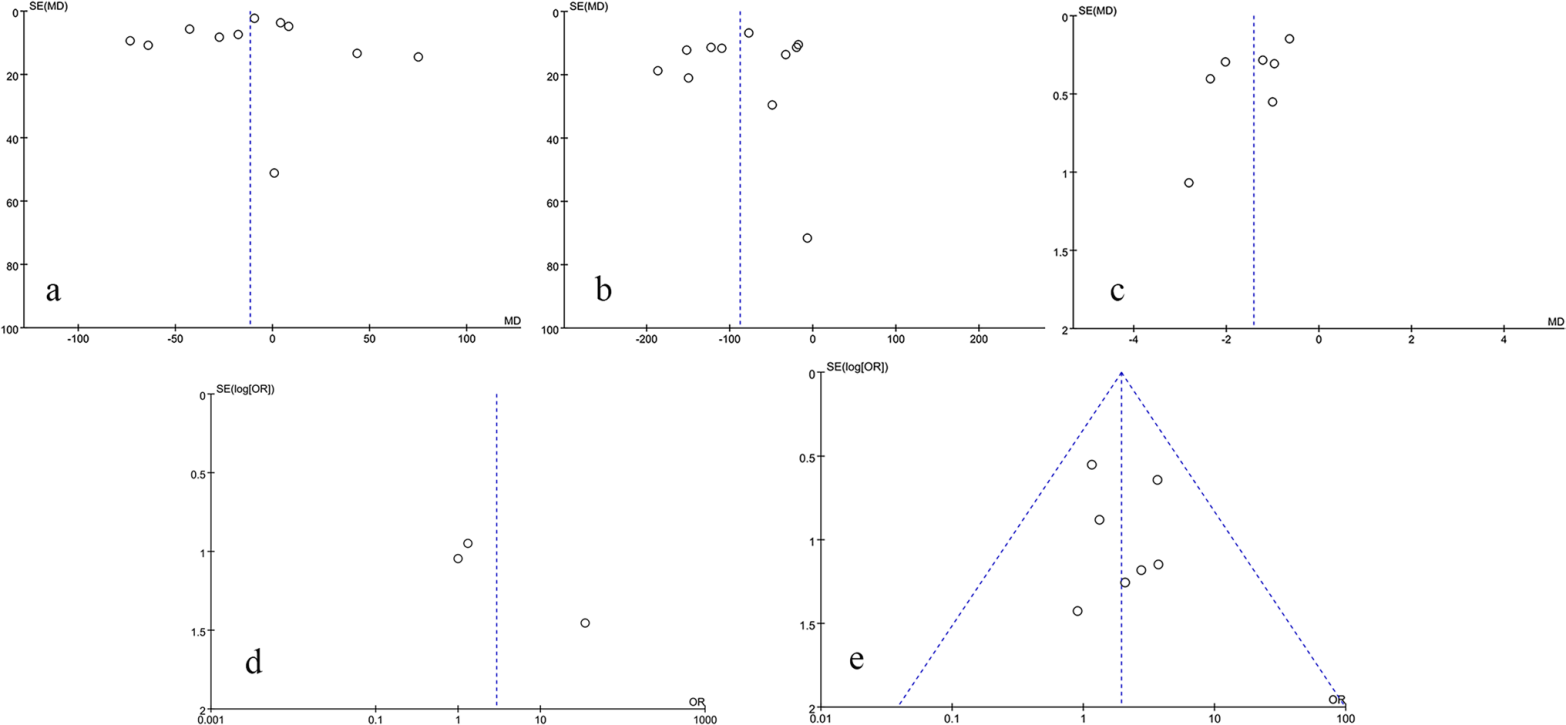
Funnel plots for evaluation of publication bias. **a** Surgery time. **b** Blood loss. **c** Hospital stay. **d** Patient satisfaction. **e** Postoperative fusion rate

**Fig. 16:**
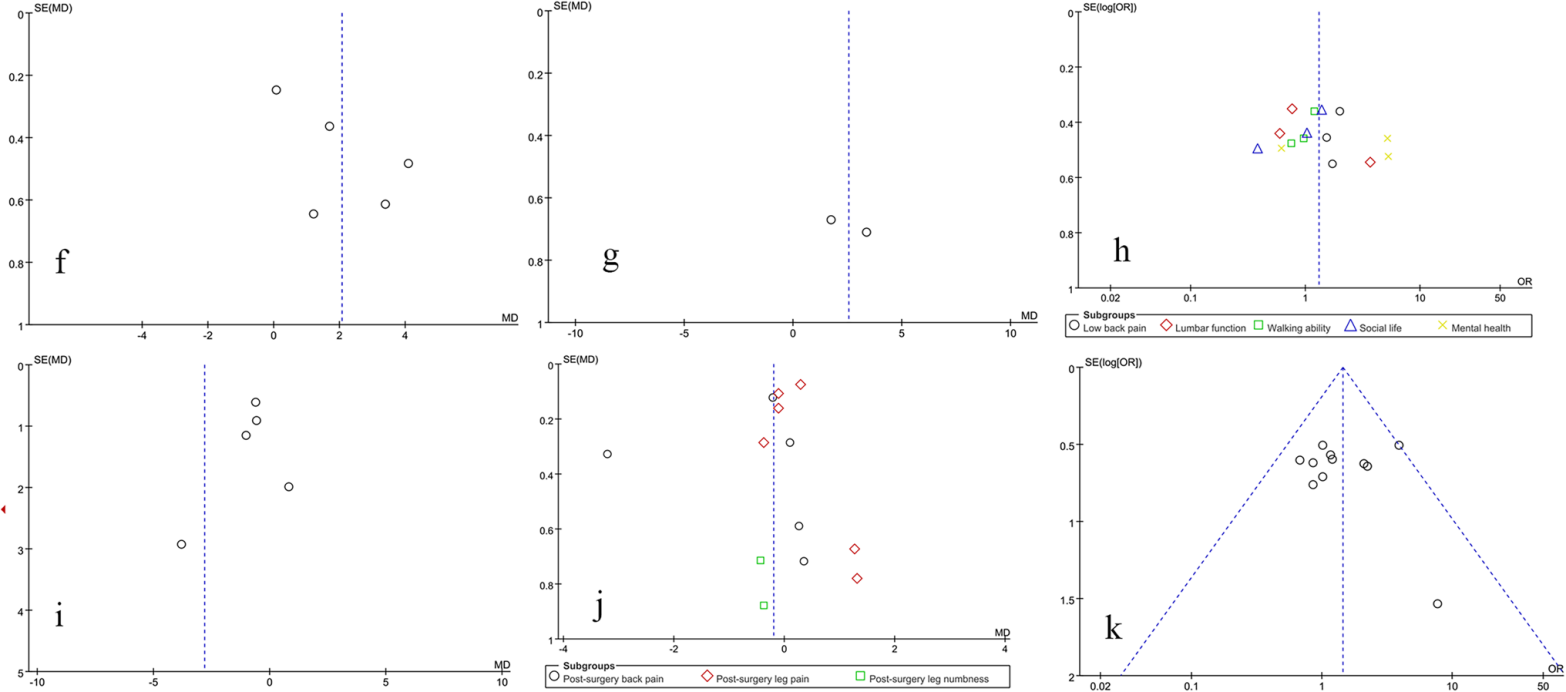
Funnel plots for evaluation of publication bias. **f** Postoperative DH. **g** Postoperative FH. **h** Postoperative JOA score. **i** Postoperative ODI function score. **j** Postoperative VAS score. **k** Postoperative complications

RevMan 5.4 software was used to assess publication bias and sensitivity of 11 outcome indicators: surgery time, blood loss, hospital stay, patient satisfaction, postoperative fusion rate, postoperative DH and FH, as well as postoperative JOA, ODI, and VAS scores, and complications. The results show that the funnel plots were symmetrical, indicating that there was no obvious publication bias and that the data were stable and reliable (Figs. 13, 14, 15, and 16).

## Discussion

As the average life expectancy increases, degenerative diseases of the lumbar spine, including spondylolisthesis, disc degeneration, and spinal stenosis, have become more common worldwide [23]. Degenerative diseases of the lumbar spine can cause lower back pain, numbness, pain in the lower extremities, lameness, and even disability, all of which can negatively affect a patient’s body. Surgical treatment is suitable for patients with poor conservative outcomes. With the advancements in medical and nursing technologies, an increasing number of patients are willing to undergo surgery. Spinal fusion, first proposed by Albee and Hibbsin [24], has gradually become the standard treatment for symptomatic lumbar degenerative diseases. A traditional surgical method is mainly an open approach, but a large number of studies have found that iatrogenic paraspinal muscle injury and other approach-related complications are drawbacks of traditional open-approach surgery [25]. With the development of minimally invasive techniques, there are now many different minimally invasive surgical modalities for surgeons to choose from. OLIF and MIS-TLIF are often used to treat degenerative lumbar spine diseases [12]. Studies have shown that OLIF can achieve indirect decompression, preserve the structure of the posterior column, correct coronal and sagittal imbalances, reduce paraspinal muscle injury, shorten surgery time, reduce perioperative blood loss, and shorten hospital stay [26]. Some studies report that MIS-TLIF is a modified version of MIS for TLIF that can directly decompress neural structures and provide good clinical and radiological outcomes [27].

This study found that the OLIF group had less intraoperative blood loss than the MIS-TLIF group. Zhu et al. [22] suggested that this may be because OLIF utilizes the natural space to reach the lumbar spine and achieves indirect decompression through DH repair. This avoids osteotomy and does not damage the posterior structures of the lumbar spine and paravertebral muscles, thereby reducing intraoperative blood loss. However, Lv et al. [28] believe that MIS-TLIF still requires open incisions, including partial paravertebral muscle tissue separation, partial laminectomy, and facet joint resection, which inevitably damage the paravertebral muscles and pose a risk of spinal instability, resulting in increased intraoperative blood loss and a prolonged hospital stay [29]. Gao et al. [13] conducted a case–control study and found that OLIF did not affect the function of the lumbar vertebral joints. Therefore, it could restore physical activity and perform the functional exercise more quickly, which is consistent with the results of this study.

Regarding the postoperative fusion rates of the two surgical methods, this meta-analysis showed that the postoperative fusion rate in the OLIF group was 90.75%, which was significantly higher than that in the MIS-TLIF group (85.55%). This is inconsistent with the results reported by Zhang et al. [30]. Studies have shown that a large-volume cage can not only provide a more efficient biological environment for the fusion process but also provide sufficient pressure. However, sufficient stress stimulation between the cage and the endplate facilitates the fusion process [31]. Simultaneously, the relatively anterior position of the cage in the OLIF group helped provide better fusion rates by correcting the sagittal imbalance, reducing endplate damage, and providing better mechanical support [32]. Contrastingly, MIS-TLIF requires the insertion of an intervertebral cage under endoscopic guidance, and the traditional cage is too bulky to pass through the working sleeve; therefore, a smaller cage must be used. This increases the risk of nonunion and cage subsidence, particularly in patients with severe osteoporosis [28]. This may explain why the fusion rate after MIS-TLIF was slightly lower than that after OLIF.

Postoperative intervertebral DH and FH recovery was better in the OLIF group than in the MIS-TLIF group. In a case–control study, Han et al. [14] found that the OLIF group had a large cage with a certain inclination implanted in the intervertebral space. A larger cage can rest on the hard epiphyseal ring around the vertebral body, thereby restoring the height of the intervertebral discs and foramen for indirect decompression. In contrast, in the MIS-TLIF group, only small cages could be implanted through a narrow surgical space. Therefore, the OLIF group could better restore DH and FH.

Both direct and indirect decompression has been shown to improve postoperative outcomes [27], which is consistent with the results of this study. Both OLIF and MIS-TLIF improved the postoperative ODI and VAS scores, but there was no significant difference between the two groups. In terms of the JOA back pain improvement rate, OLIF was superior to MIS-TLIF. Research has shown that the back muscles play a vital role in connecting several major muscles in the body [33]. OLIF allows the back muscles to remain intact postoperatively. Although MIS-TLIF is an improvement on TLIF technology, the use of a tubular retractor through Wiltse’s approach is undoubtedly one of the means to preserve the back muscles [34]. However, it still inevitably damages the paraspinal muscles [35]. This may be one of the reasons why OLIF is superior to MIS-TLIF for enhancing the back pain improvement rate.

In this meta-analysis, we concluded that the complication rates were 18.1% and 13.6% in the OLIF and MIS-TLIF groups, respectively. Dural tears and nerve root injuries due to stenosis of the transforaminal passage are the most common complications associated with MIS-TLIF due to stenosis of the transforaminal passage [36]. Contrastingly, segmental artery injury and transient thigh numbness were common complications in the OLIF group because the lumbar plexus, lumbar sympathetic trunk, and segmental arteries are all located anterior to the lumbar spine and are susceptible to irritation or injury [37]. The abdominal aorta is on the left anterolateral side of the lumbar spine, and the vena cava is on the right anterolateral side of the lumbar spine. At the same time, vascular tissue, especially in the venous system, has various anatomical variations. The early branches of the internal iliac vein are typical variants that interfere with OLIF [17]. The ureter is located behind the peritoneum and descends vertically through the medial anterior part of the psoas muscle into the pelvis. The ureter is susceptible to injury at any stage of the retroperitoneal passage anatomy and during tubular retractor placement [38]. According to Javier et al. [39], 90.4% of ureteral injuries were anterior to the psoas muscle, and 16% were lateral to the vertebral body. This may be due to the orthopedic surgeon’s unfamiliarity with the retroperitoneal structure. Overall, the OLIF group had a higher complication rate than the MIS-TLIF group.

The results of this meta-analysis showed that, compared with the MIS-TLIF group, the OLIF group had the advantages of lower blood loss, a shorter hospital stay, a higher postoperative fusion rate, and better intervertebral DH and FH recovery. However, MIS-TLIF is associated with a relatively low complication rate. The postoperative functional scores of the two groups were roughly the same; however, the OLIF group had better JOA score improvement in back pain than the MIS-TLIF group, and there was no significant difference in surgery time or patient satisfaction.

This meta-analysis has the following shortcomings. (1) A total of 13 studies were included in this meta-analysis, of which there were insufficient randomized controlled trials and the level of evidence was moderate; (2) the surgical staffs of OLIF and MIS-TLIF in different studies were different, which may have affected the surgical effect; (3) among the outcome indicators, 11 articles were included in the same indicator and at least three articles were included, increasing the heterogeneity among studies slightly; and (4) some preoperative indicators (like disease severity) for the two groups of patients lacked accurate data in the included literature. Because this meta-analysis was based on secondary literature, statistical analyses could not be carried out. Due to certain limitations and biases in this meta-analysis, the reliability of the results may have been reduced. Therefore, a large number of rigorous studies with large sample sizes, multicenter studies, correct randomization principles, blinding, allocation concealment, and other literature studies are needed to further demonstrate this.

## Data Availability

The datasets supporting the conclusions of this article are included within the article (and its additional files).
